# Supplementary material for: Complexome profiling of the Chlamydomonas psb28 mutant reveals TEF5 as an early PSII assembly factor
Source: Plant Cell. 2025 Mar 17;37(6):koaf055. doi: 10.1093/plcell/koaf055 (PMC12164590; doi:10.1093/plcell/koaf055)
Supplement: koaf055_Supplementary_Data [file koaf055_supplementary_data.zip › psb28_interactive.zip!/plots/intensity/qualityPlots/peptides/PRPL15_Cre14.g612450.t1.2.html]

### Cre14.g612450.t1.2

Trivial name: PRPL15  
SeqCoverage [median]: 19.50  
Mass: 26.04 kDa  
  
 

| Sequence | MissedCleavage | Mass [Da] | wtIntensity | muIntensity | Score |
| --- | --- | --- | --- | --- | --- |
| DASLPLKVLGTGSLSK | 1 | 1584.91 | 520.73 | 0.00 | 45.86 |
| SGPSVRPGFEGGQTPLYR | 1 | 1903.95 | 0.00 | 1174.61 | 68.13 |
| SLNVHATAFSESAK | 0 | 1460.73 | 0.00 | 545.34 | 77.53 |
|  |  |  | 520.7 | 1719.9 |  |
